# Clinical and radiological outcomes in patients who underwent posterior lumbar interbody fusion: comparisons between unilateral and bilateral cage insertion

**DOI:** 10.1186/s12891-021-04852-y

**Published:** 2021-11-17

**Authors:** Jae Hwan Cho, Chang Ju Hwang, Dong-Ho Lee, Choon Sung Lee

**Affiliations:** Department of Orthopedic Surgery, Asan Medical Center, University of Ulsan College of Medicine, 388-1, PungNap-2-dong, SongPa-gu, Seoul, South Korea

**Keywords:** Posterior lumbar interbody fusion, Cage, Outcomes, Cohort matching, Unilateral, Bilateral, Pseudarthrosis, Risk factor

## Abstract

**Background:**

Although the original technique involves inserting two cages bilaterally, there could be situations that only allow for insertion of one cage unilaterally. However, only a few studies have compared the outcomes between unilateral and bilateral cage insertion. The purpose of this study was to compare the clinical and radiological outcomes in patients who underwent posterior lumbar interbody fusion (PLIF) between unilaterally and bilaterally inserted cages.

**Methods:**

Among 206 eligible patients who underwent 1- or 2-level PLIF, 78 patients were 1:3 cohort-matched by age, sex, and operation level (group U, 19 patients with unilateral cages; and group B, 57 patients with bilateral cages). Fusion status was evaluated by computed tomography (CT) scans at postoperative 1 year. Clinical outcomes were measured by visual analog scale (VAS), Oswestry Disability Index (ODI), and EQ-5D. Radiological and clinical parameters were compared between the two groups. Risk factors for pseudarthrosis were also analyzed by multivariate analysis.

**Results:**

The demographic data were not significantly different between the two groups. However, previous laminectomy, asymmetric disc collapse, and fusion at L5-S1 level were more frequently found in group U (*P* = 0.003, *P* = 0.014, and P = 0.014, respectively). Furthermore, pseudarthrosis was more frequently observed in group U (36.8%) than in group B (7.0%) (*P* = 0.004). Back pain VAS was higher in group U at postoperative 1 year (*P* = 0.033). Lower general activity function of EQ-5D was observed in group U at postoperative 1 year (*P* = 0.035). Older age (*P* = 0.028), unilateral cage (*P* = 0.007), and higher bone mineral density (*P* = 0.033) were positively correlated with pseudarthrosis.

**Conclusions:**

Unilaterally inserted cage might be a possible risk factor for pseudarthrosis when performing PLIF, which could be related with the difficult working conditions such as scars due to previous laminectomy or asymmetric disc collapse. Furthermore, suboptimal clinical outcomes are expected following PLIF with unilateral cage insertion at postoperative 1 year regardless of similar clinical outcomes at postoperative 2 year. Therefore, caution is advised when inserting cages unilaterally, especially under above-mentioned conditions in terms of its possible relationship with symptomatic pseudarthrosis.

## Background

Posterior lumbar interbody fusion (PLIF) is a well-proven procedure to perform in patients who require stabilization following wide decompression for spinal stenosis or spondylolisthesis [1, 2]. PLIF involves cage insertion through the disc space. Although two cages are inserted bilaterally in the original technique, situations can arise in which only one cage can be inserted unilaterally, such as in severe asymmetric disc space narrowing, severe adhesion due to previous laminectomy or discectomy, or anatomic root variation [3]. In the above cases, unilateral cage insertion, posterolateral fusion, or anterior lumbar interbody fusion could be considered as an alternative [4].

Several previous studies have compared the clinical and radiological outcomes of unilaterally and bilaterally inserted cages. A few studies have revealed the effectiveness of unilateral interbody cage insertion in terms of similar clinical and radiological outcomes compared to that of bilateral cage insertion if bilateral screw fixation was conducted [5, 6].

However, contrary to the previous belief, we experienced frequent pseudarthrosis when the cage was unilaterally inserted in the PLIF procedure. Thus, our basic hypothesis was that the postoperative fusion rate in cases with unilaterally inserted cages is inferior to those with bilateral cages. The main purpose of this study was to compare the clinical and radiological outcomes in patients who underwent PLIF between unilateral and bilateral cage groups.

## Methods

### Patients and operative methods

Among 206 consecutive, eligible patients who underwent 1- or 2-level PLIF for spinal stenosis or spondylolisthesis from March 2014 to January 2018 in our institution, a total of 78 patients were finally included in this study. Because of an unbalanced ratio between unilateral (*n* = 19) and bilateral (*n* = 187) cage insertion, 1:3 cohorts were matched by age, sex, and operation level (group U, 19 patients with unilateral cages; and group B, 57 patients with bilateral cages). The indication of PLIF was as follows: (1) spondylolisthesis, (2) symptomatic neural foraminal stenosis, (3) combined severe back pain because of severe facet degeneration.

All surgeries were performed by a single surgeon, and patients were followed up for more than 2 years. The operative procedure was conducted in a routine manner as follows: bilateral pedicle screw insertions, subtotal laminectomy, bilateral facetectomy, disc preparation, packing of local bone fragments in the anterior intervertebral space and PEEK (polyetheretherketone) cage insertion with bone graft material (local bone fragments with DBM or BMP-2). We did not experience the lack of bone graft even in revisional surgeries because we could get the morselized bone from additional laminectomy and medial total facetectomy. We usually tried to insert cages bilaterally, and we only inserted a cage unilaterally in specific conditions, such as in cases with a narrow working space or aberrant nerve root.

### Study variables

Demographic data and operation-related data were obtained by electronic chart reviews. The visual analog scale (VAS), Oswestry Disability Index (ODI), and EuroQol 5-dimension questionnaire (EQ-5D) were used to evaluate clinical outcomes. The length of stay, operation time, estimated blood loss, bone graft material, and postoperative complications were also reviewed. Fusion status was evaluated by three-dimensional computed tomography (CT) scans at postoperative 1-year [7]. We defined bony fusion as existence of a bridging trabecular bone, mature bony trabeculae bridging the interbody space, or cortication at the peripheral edges of fusion masses between both endplates, and without cystic radiolucency in coronal and sagittal reconstructed images of thin-section CT scans [8]. If pseudarthrosis was found only one level in 2-level fusion cases, then this case was regarded as pseudarthrosis. If there was evidence of solid fusion for only one cage in bilaterally inserted cases, then this case was regarded as fusion. All patients were followed up at 1, 3, 6, and 12 months postoperatively, and yearly thereafter. This study was approved by our Institutional Review Board, which waived the requirement for informed consent given the retrospective nature of the analysis.

### Statistical analyses

Demographic data, operation-related data, and clinical outcomes were compared between group U and group B by either the Student’s t-test or chi-square test. Multivariate logistic regression analysis was performed to control for intervening factors. Further analysis in accordance with fusion status at 1-year postoperative was performed. Statistical analyses were performed using the Statistical Package for Social Sciences software, version 21.0 (SPSS, Chicago, IL), with *P*-values < 0.05 considered to indicate statistical significance.

## Results

The mean age of the participants was 65.4 years old, and the sex ratio was 10:9 (male:female). The ratio of operation levels was 9:10 (1 vs 2 level). There were no significant differences in demographic data between the two groups, with the exception of operation level (Table 1). We could insert cages bilaterally more easily at L4–5 level or above (77.2% for group B and 42.1% for group U). However, 56.9% (11/19) of group U included L5–S1 level.

**Table 1:** Demographic data of age, sex, operation level-matched cohort

	Category	Group U (*N* = 19)	Group B (*N* = 57)	*P* value
Age (yr)		65.6 ± 10.7	65.3 ± 9.0	0.905
Sex	M	10 (52.6%)	30 (52.6%)	1.000
	F	9 (47.4%)	27 (47.4%)	
Height (cm)		160.0 ± 8.7	156.4 ± 8.8	0.125
Weight (kg)		66.4 ± 9.9	61.4 ± 9.5	0.053
BMI (kg/m^2^)		25.9 ± 2.4	25.0 ± 2.7	0.229
BMD (T score)		−0.6 ± 1.6	− 0.9 ± 1.3	0.515
Smoking	Y	5 (26.3%)	6 (10.5%)	0.090
	N	14 (73.7%)	51 (89.5%)	
Number of Op. level	1	9 (47.4%)	27 (47.4%)	1.000
	2	10 (52.6%)	30 (52.6%)	
Op. level	L3–4	1 (5.3%)	3 (5.3%)	0.014
	L3–4-5	5 (26.3%)	20 (35.1%)	
	L4–5	2 (10.5%)	21 (36.8%)	
	L4–5-S1	5 (26.3%)	10 (17.5%)	
	L5-S1	6 (31.6%)	3 (5.3%)	
Previous laminectomy	Yes	5 (26.3%)	1 (1.8%)	0.003
Asymmetric disc collapse^a^	**Yes**	**6 (31.5%)**	**5 (8.8%)**	**0.014**

Mean and standard variation in continuous variables and number of cases in categorical variables

Group U: Unilateral cage insertion; Group B: Bilateral cage insertion

*M* Male, *F* Female, *BMI* Body mass index, *BMD* Bone mineral density

^a^ Five or more degree of asymmetric disc wedging on the radiographs

The reasons for unilateral cage insertion were as follows: Eight asymmetric disc collapse, six severe adhesion, three suspicious nerve root variation, and two other reasons.

### Comparisons of clinical and radiological outcomes between group U and group B

There were no significant differences in hospital stay, operation time, and estimated blood loss between the two groups. However, a higher fusion rate was observed in group B (93.0% in group B and 63.2% in group U), irrespective of the fact that similar bone graft material was used. In addition, more previous laminectomy at the same site and asymmetric disc wedging was found in group U (*P* = 0.003 and 0.014, respectively). Although there were four cases of screw-related complications in group B, no postoperative neurologic compromise was found in either of the groups postoperatively. We did not experience revisional surgery during 2-year follow-up period. The overall operation-related data are summarized in Table 2.

**Table 2:** Comparisons of operation related data between 2 cohorts

	Category	Group U (*N* = 19)	Group B (*N* = 57)	*P*-value
Length of stay		11.8 ± 2.3	12.0 ± 3.2	0.791
Operation time		138.7 ± 31.7	151.1 ± 36.9	0.191
Estimated blood loss		426.6 ± 305.8	528.0 ± 408.0	0.379
Bone graft material	Local bone	2 (10.5%)	8 (14.0%)	0.098
	DBM	3 (15.8%)	23 (40.4%)	
	BMP	14 (73.7%)	26 (45.6%)	
Fusion state at 1Y	Yes	12 (63.2%)	53 (93.0%)	0.004
	No	7 (36.8%)	4 (7.0%)	
Complications	Screw related	0 (0.0%)	4 (7.0%)	0.567
	Dural tear	2 (10.5%)	1 (1.8%)	0.152
	Neurologic	0 (0.0%)	0 (0.0%)	NA
	FBSS	1 (5.3%)	6 (10.5%)	0.672

Mean and standard variation in continuous variables and number of cases in categorical variables

Group U: Unilateral cage insertion; Group B: Bilateral cage insertion

*DBM* Demineralized bone matrix, *BMP* Bone morphogenetic protein, *FBSS* Failed back surgery syndrome

**Fig. 1:**
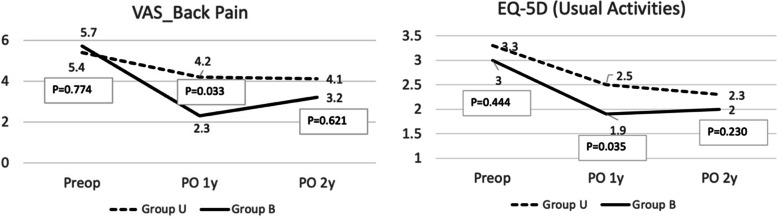
Comparisons of clinical outcomes between group U (unilateral cage) and group B (bilateral cage). **a** Visual analog scale (VAS) of back pain, and **b** usual activities domain of EQ-5D

**Fig. 2:**
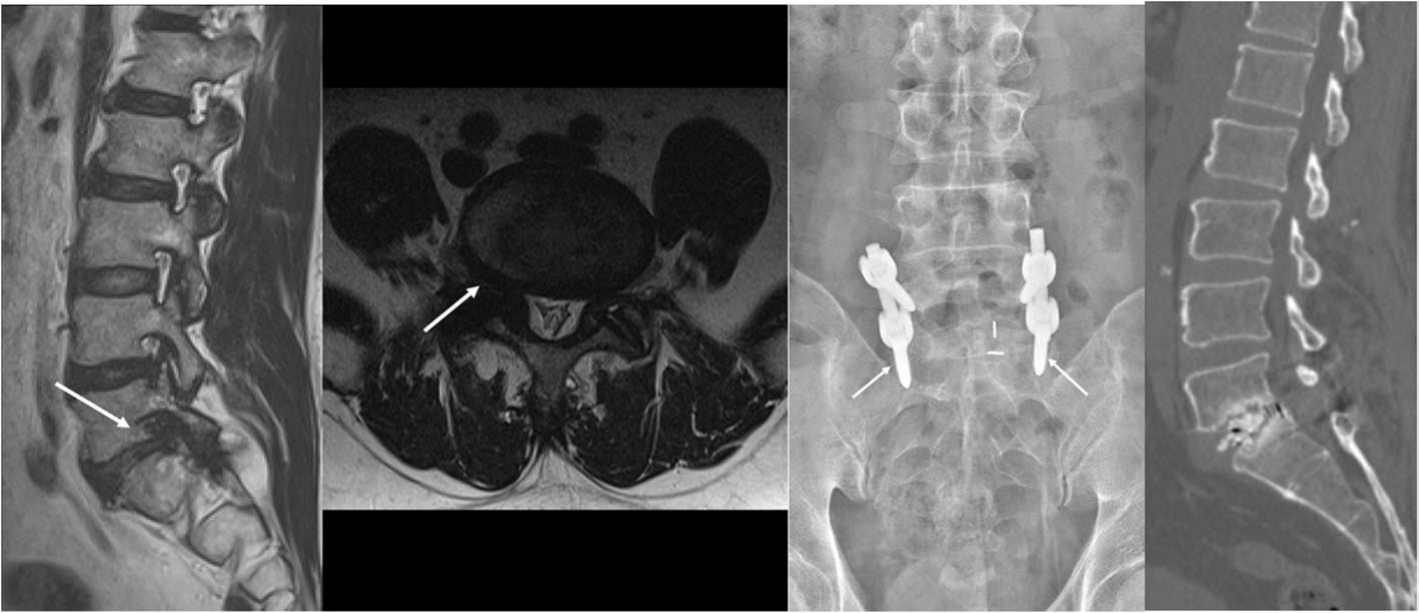
Illustrative cases showing the pseudarthrosis following unilateral cage insertion. **a** A 56-year-old male patient with right buttock pain and leg weakness (BMD T-score, − 0.3). MRI (T2-weighted sagittal and axial) showed severe right foraminal stenosis (arrows). **b** A plain radiograph at postoperative 1 year showed radiolucency around S1 screws (arrows). **c** Definite pseudarthrosis was observed in the 1-year CT sagittal reconstruction image

**Fig. 3:**
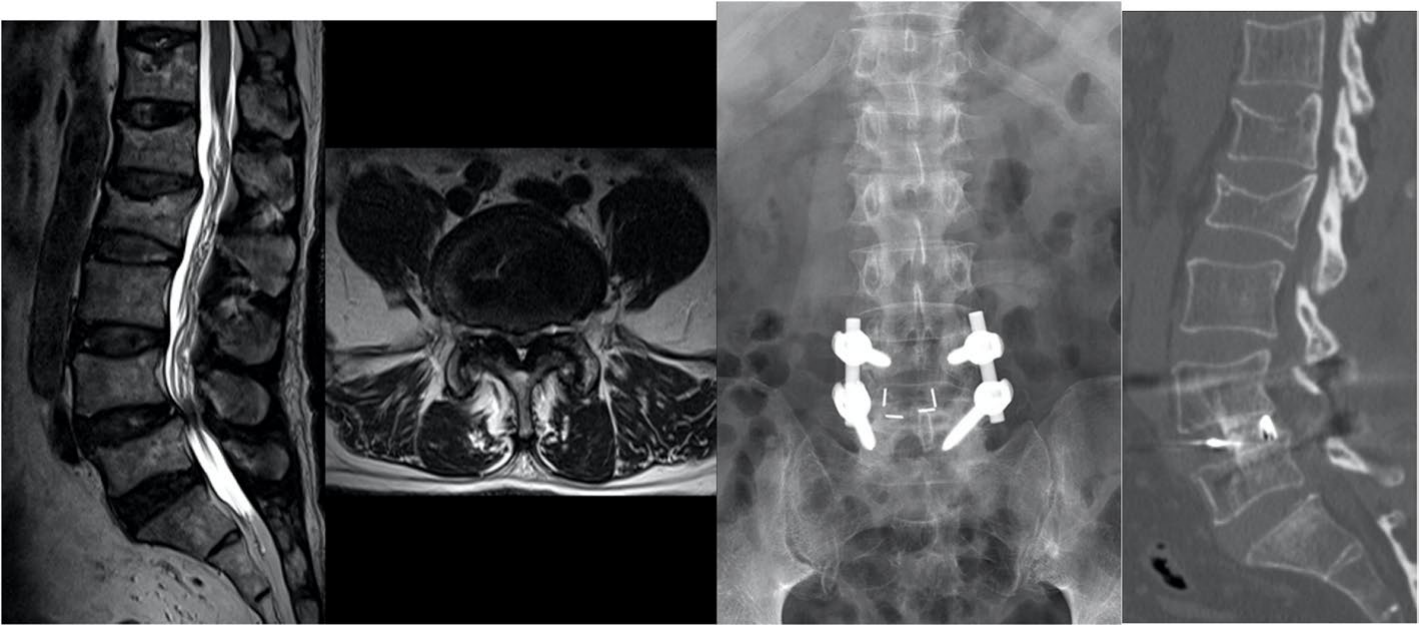
Illustrative cases showing the solid fusion following bilateral cage insertion. **a** A 55-year-old male patient with low back pain with sciatica (BMD T-score − 1.6). MRI (T2-weighted sagittal and axial) showed severe central stenosis with degenerative spondylolisthesis. **b** No radiolucency was observed in the postoperative 1-year plain radiograph. **c** Solid fusion was observed in the 1-year CT sagittal reconstruction image

The clinical and functional outcomes are described in Fig. 1 and Table 3. None of the preoperative variables showed significant differences between the two groups. The back pain VAS was significantly higher in group U than in group B at 1 year postoperative (4.2 vs 2.3, *P* = 0.033); however, this difference disappeared at the 2-year follow-up (*P* = 0.637). There were no significant differences in terms of leg pain VAS and ODI. There was a significant difference in EQ-5D (usual activities) at 1 year postoperative (2.5 in group U and 1.9 in group B, *P* = 0.035), although this change disappeared at the 2-year follow-up (*P* = 0.230). The mobility (2.4 and 1.9, *P* = 0.094) and self-care (2.0 vs 1.6, *P* = 0.083) domain of EQ-5D showed better outcomes in group B at 2 year postoperative, although this did not reach statistical significance. Representative cases are illustrated in Fig. 2 and Fig. 3.

**Table 3:** Comparisons of clinical and functional outcomes between 2 groups

	Periods	Group U (*N* = 19)	Group B (*N* = 57)	*P* value
VAS_back	Preop	5.4 ± 2.4	5.7 ± 2.7	0.774
	PO 1y	4.2 ± 3.0	2.3 ± 2.7	0.033
	PO 2y	4.1 ± 2.8	3.2 ± 3.3	0.621
VAS_leg	Preop	5.8 ± 2.4	5.9 ± 2.8	0.534
	PO 1y	2.0 ± 2.9	2.4 ± 2.6	0.637
	PO 2y	3.3 ± 3.1	3.2 ± 3.0	0.878
ODI	Preop	51.0 ± 16.1	53.0 ± 18.1	0.732
	PO 1y	30.6 ± 18.6	24.0 ± 15.3	0.213
	PO 2y	35.0 ± 18.0	28.2 ± 18.9	0.192
EQ-5D (mobility)	Preop	3.8 ± 0.9	3.4 ± 0.9	0.154
	PO 1y	2.2 ± 1.0	2.0 ± 0.9	0.380
	PO 2y	2.4 ± 1.0	1.9 ± 0.9	0.094
EQ-5D (self-care)	Preop	2.6 ± 0.9	2.6 ± 1.0	0.982
	PO 1y	2.0 ± 1.0	1.6 ± 0.8	0.135
	PO 2y	2.0 ± 0.7	1.6 ± 0.8	0.083
EQ-5D (usual activities)	Preop	3.3 ± 1.1	3.0 ± 0.9	0.444
	PO 1y	2.5 ± 0.9	1.9 ± 0.9	0.035
	PO 2y	2.3 ± 1.0	2.0 ± 0.8	0.230
EQ-5D (pain/discomfort)	Preop	3.6 ± 0.9	3.5 ± 1.0	0.748
	PO 1y	2.8 ± 1.0	2.4 ± 0.7	0.126
	PO 2y	2.7 ± 0.7	2.4 ± 0.9	0.150
EQ-5D (anxiety/depression)	Preop	2.4 ± 1.0	2.4 ± 1.1	0.996
	PO 1y	1.7 ± 1.0	1.6 ± 0.8	0.823
	PO 2y	1.8 ± 0.9	1.7 ± 0.9	0.663

*VAS* Visual analogue scale, *ODI* Oswestry disability index, *EQ-5D* EuroQol 5-dimension questionnaire

### Comparisons of various parameters in accordance with fusion status

The fusion status at postoperative 1 year was associated with the number of cage insertions. The odds ratio for development of pseudarthrosis between group U compared to group B was 7.73 (95% confidence interval [1.95, 30.69]). Only 1 pseudarthrosis case in 2-level PLIF cases showed partial union in one level (L5-S1) and pseudarthrosis in the other level (L4–5). We could not find any partial fusion case (solid fusion in one cage and pseudarthrosis in the other cage) in group B. In addition, younger age (63.8 vs 70.1 years, *P* = 0.028) and relatively lower bone mineral density (BMD) (− 1.0 vs 0.2, *P* = 0.033) were associated with postoperative bony fusion. However, sex, body mass index, bone graft material, previous laminectomy, radiological asymmetric disc wedging, operation level, and smoking were not associated with fusion (Table 4).

**Table 4:** Comparative analysis by the fused status at postoperative 1-year

	Category	Fusion (*N* = 65)	Pseudarthrosis (*N* = 11)	*P*-value (Uni)	*P*-value (Multi)	Beta	SE
Age		63.8 ± 9.3	70.1 ± 9.7	0.041	0.028	0.138	0.063
Sex	M	24 (36.9%)	6 (54.5%)	0.269	NA	NA	NA
	F	41 (63.1%)	5 (45.5%)				
Cages	Unilateral	12 (18.5%)	7 (63.6%)	0.004	0.007	2.429	0.901
	Bilateral	53 (81.5%)	4 (36.4%)				
BMI		25.0 ± 2.4	27.0 ± 3.2	0.019	0.069	0.333	0.183
BMD		−1.0 ± 1.2	0.2 ± 1.7	0.006	0.033	0.708	0.332
Bone graft material	Local bone	8 (12.3%)	1 (9.1%)	0.759	NA	NA	NA
	DBM	23 (35.4%)	3 (27.3%)				
	BMP	33 (50.8%)	7 (63.6%)				
Previous laminectomy	Y N	4 (7.4%) 61 (92.6%)	2 (18.2%) 9 (91.8%)	0.207	NA	NA	NA
Asymmetric disc wedging^a^	Y N	9 (13.8%) 56 (86.2%)	2 (18.2%) 9 (91.8%)	0.656	NA	NA	NA
Number of Op. level		1.58 ± 0.50	1.55 ± 0.52	0.811	NA	NA	NA
Level	L3–4	2 (3.1%)	1 (9.1%)	0.890	NA	NA	NA
	L3–4-5	23 (35.4%)	3 (27.3%)				
	L4–5	12 (18.5%)	2 (18.2%)				
	L4–5-S1	15 (23.1%)	3 (27.3%)				
	L5-S1	13 (20.0%)	2 (18.2%)				
Smoking	Y	9 (13.8%)	2 (18.2%)	0.656	NA	NA	NA
	N	56 (86.2%)	9 (81.8%)				

Mean and standard variation in continuous variables and number of cases in categorical variables

*M* Male, *F* Female, *BMI* Body mass index, *BMD* Bone mineral density, *DBM* Demineralized bone matrix, *BMP* Bone morphogenetic protein

^a^ Five or more degree of asymmetric disc wedging on the radiographs

## Discussion

The PLIF procedure is traditionally used to enhance intervertebral stability following decompression for spinal stenosis [9]. Initially, unicortical or bicortical bone grafts were inserted in both sides to enhance interbody fusion [10, 11]. The use of cages was reported for the first time in 1993 [12], and cages have been used successfully since then [13, 14]. Although cages were usually inserted bilaterally, the effectiveness of using unilateral cages has been also suggested, and comparable clinical outcomes and fusion rates have been observed in patients who underwent unilateral cage insertion.

We generally used bilateral cages in PLIF based on the belief that they have superior biomechanical stability, which could lead to better clinical and radiological outcomes. In one study, a higher immediate stability was proposed when PLIF was performed with bilateral cages compared to transforaminal lumbar interbody fusion (TLIF) [15]. Because the cage in TLIF has a wider surface than that in PLIF, the difference between unilateral and bilateral cages is probably greater in cases with PLIF. However, clinical studies have shown favorable results using single cages in an instrumented fusion. In one study, the rate of dural tear was higher in the bilateral cage group [16]. Shorter operative time and less blood loss in cases of two level fusion was also reported as an advantage of single cages [5]. However, few studies have revealed the superiority of clinical and radiological outcomes with single cage compared to with bilateral cages.

Recently, TLIF with a single cage is frequently considered because it has an advantage of less blood loss and shorter operative time. However, sometimes it is difficult to insert a cage with a larger surface area for TLIF in cases with a narrow disc space. In this case, it is easier to perform PLIF with smaller cages. However, there are certain conditions that prevent cage insertion. For example, root variation, such as dual root, severe fibrosis due to previous surgery, or failure to distraction of the narrow disc space because of severe disc degenerative changes are probable obstacles to prevent insertion of cages. In the above-mentioned situations, unilateral cage insertion was tried instead of posterolateral fusion (PLF) in this study due to the risk of sagittal malalignment.

Importantly, the union rate was inferior in unilateral cage group but not in the bilateral cage group (63.2% vs 93.0%) in the current study. The main reasons of this inferior fusion rate are thought to be (1) subsidence following over-distraction in cases with narrow disc spaces; (2) stress concentration on the cage as a result of asymmetric disc space on the coronal plane, such as degenerative scoliosis; and (3) a relatively small contacting area between endplates by the cage. More previous laminectomy, asymmetric disc wedging, and L5-S1 level were frequently found in group U, the effect of those factors on pseudarthrosis were not revealed (Table 4). However, above-mentioned conditions could be risk factors of nonunion [17, 18], although the risk factors of nonunion have not been clearly defined until now. In general, those factors are regarded as difficult conditions for performing spinal surgery. The reason of higher proportion of L5-S1 in group U is not clear. However, frequently found unilateral disc collapse at L5-S1 might be one of the reasons, which could make it hard to insert a cage in severely collapsed side. Failure to reveal the effect of high proportion of L5-S1 in group U on pseudarthrosis is thought to be small sample size, which could be actual reason of inferior outcomes at postoperative 1-year. In this regard, difficult conditions to performing cage insertion such as limited bone stock due to previous laminectomy and disc space collapse at L5-S1 level could be more important factors. However, this could not be revealed by our study due to small sample size in group U.

The effect of nonunion on the postoperative symptoms also has been very controversial. Although back pain VAS and usual activity domain of EQ-5D showed inferior outcomes in postoperative 1 yr in group U, this difference disappeared in 2-yr follow-up. Many confounding factors could involve in this result, which made it difficult to interpret the result. In the past, many studies proposed no differences of clinical outcomes regardless of fusion status postoperatively [19, 20]. However, a recent meta-analysis showed a superior result of the fused group in both clinical and functional outcomes [21]. This finding explains the increased back pain and lower functional scores in patients who underwent PLIF with unilateral cages, as well as the higher pseudarthrosis rate. However, many studies comparing TLIF with PLIF showed no differences in clinical and radiological outcomes, although fewer complication rates were noted in patients with TLIF [22, 23]. The comparable fusion rate with TLIF could be explained by the wider contact area of the cage used in TLIF.

Interestingly, we found two additional independent risk factors for pseudarthrosis: older age and higher BMD. The relationship between age and nonunion was also supported by one previous study, although many studies found no relationship between age and fusion rate [24, 25]. However, a few previous studies suggested that osteoporosis is related to a higher rate of nonunion, which disputed our results, although many other studies also denied the relationship between BMD and nonunion [18, 26]. However, we think the more degenerative process such as sclerotic change could be related with the disc degeneration and collapse, which means the difficulty of cage insertion. Sometimes, bone density was checked to be higher due to the severe degeneration [27]. So, we did not think higher BMD itself is not the real cause of pseudarthrosis. In this regard, thorough investigation with a larger sample size might reveal the effect of demographic variables, such as age and BMD on the radiological outcome.

There are a few limitations to this retrospective study. First, the number of patients in each group was unbalanced, because we always tried to insert cages bilaterally, except in specific conditions. As a result, we performed 1:3 cohort matching to minimize the selection bias. Second, defining fusion status by CT is controversial; although many studies use CT to check the fusion status postoperatively, this method also has type I and type II errors, and is subjective. However, the diagnostic accuracy of CT to check pseudarthrosis is reported to be superior to that of lumbar radiographs [28]. Third, we could not reveal the previously known risk factors for nonunion, such as smoking, because of the relatively small sample size. Fourth, we did not check routine 2-year CT to analyzed fusion status, which made it impossible to analyze the effect of fusion rate on clinical outcomes in postoperative 2-year. However, we believed this study reflected the real clinical setting such as unavoidable unilateral cage insertion by various conditions.

## Conclusions

In conclusion, unilaterally inserted cage might be a possible risk factor for pseudarthrosis when performing PLIF, which could be related with the difficult working conditions such as scars due to previous laminectomy or asymmetric disc collapse. Furthermore, suboptimal clinical outcomes are expected following PLIF with unilateral cage insertion at postoperative 1 year regardless of similar clinical outcomes at postoperative 2 year. Therefore, caution is advised when inserting cages unilaterally, especially under above-mentioned conditions in terms of its possible relationship with symptomatic pseudarthrosis.

## Data Availability

The datasets used and/or analysed during the current study are not publicly available due to limitations of ethical approval involving the patient data and anonymity but are available from the corresponding author on reasonable request.

## Abbreviations

PLIF: Posterior lumbar interbody fusion
VAS: Visual analog scale
ODI: Oswestry Disability Index
EQ-5D: EuroQol 5-dimension questionnaire
CT: Computed tomography
BMD: Bone mineral density
TLIF: Transforaminal lumbar interbody fusion
